# Supplementary figures and images for: Effect of tryptophan starvation on inclusion membrane composition and chlamydial-host interactions
Source: Infect Immun. 2025 Jan 13;93(2):e00532-24. doi: 10.1128/iai.00532-24 (PMC11834466; doi:10.1128/iai.00532-24)

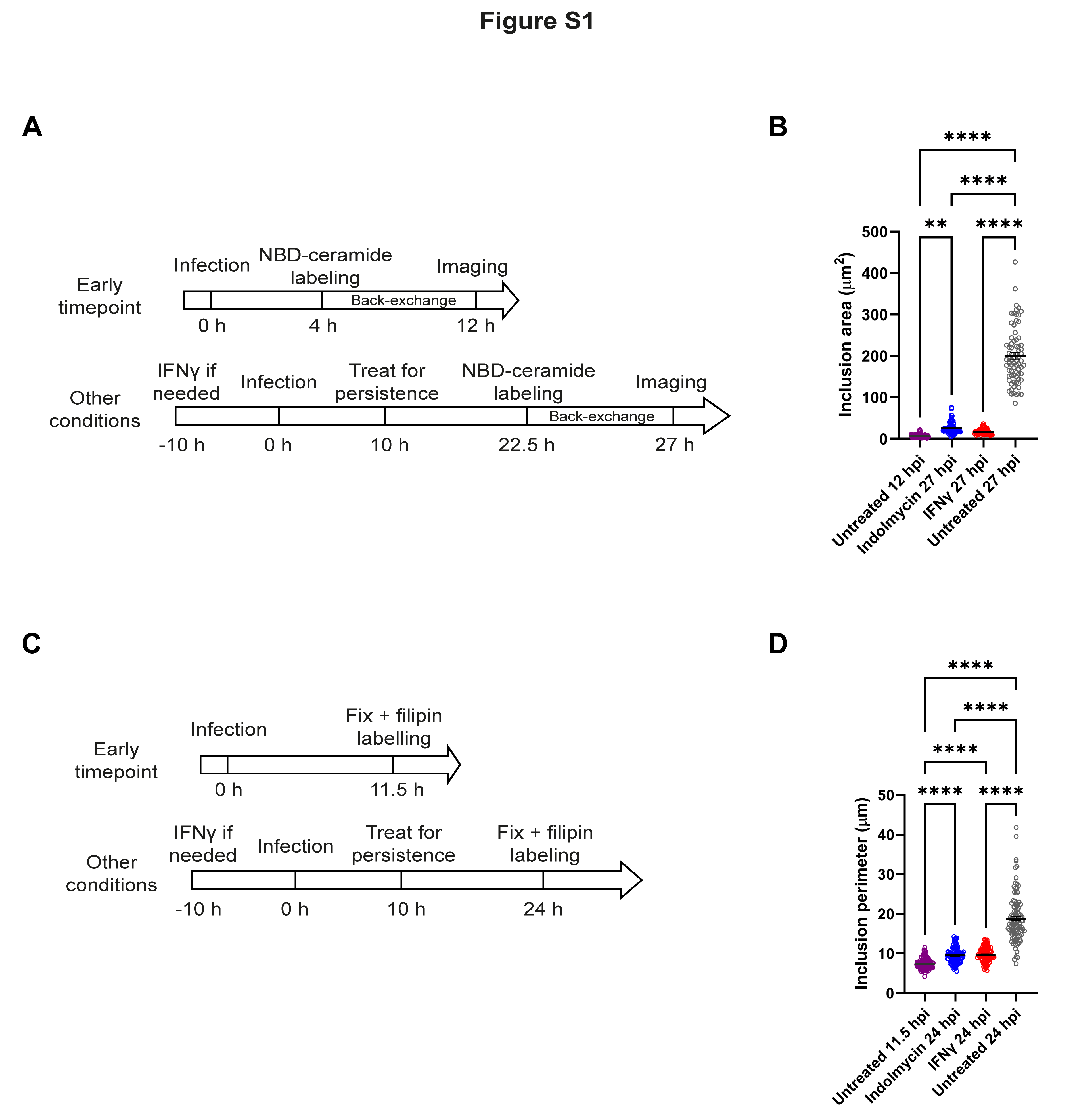

Supplement: Fig. S1 — Associated data for NBD-SM and cholesterol data, Fig. 1. [file iai.00532-24-s0001.tif]

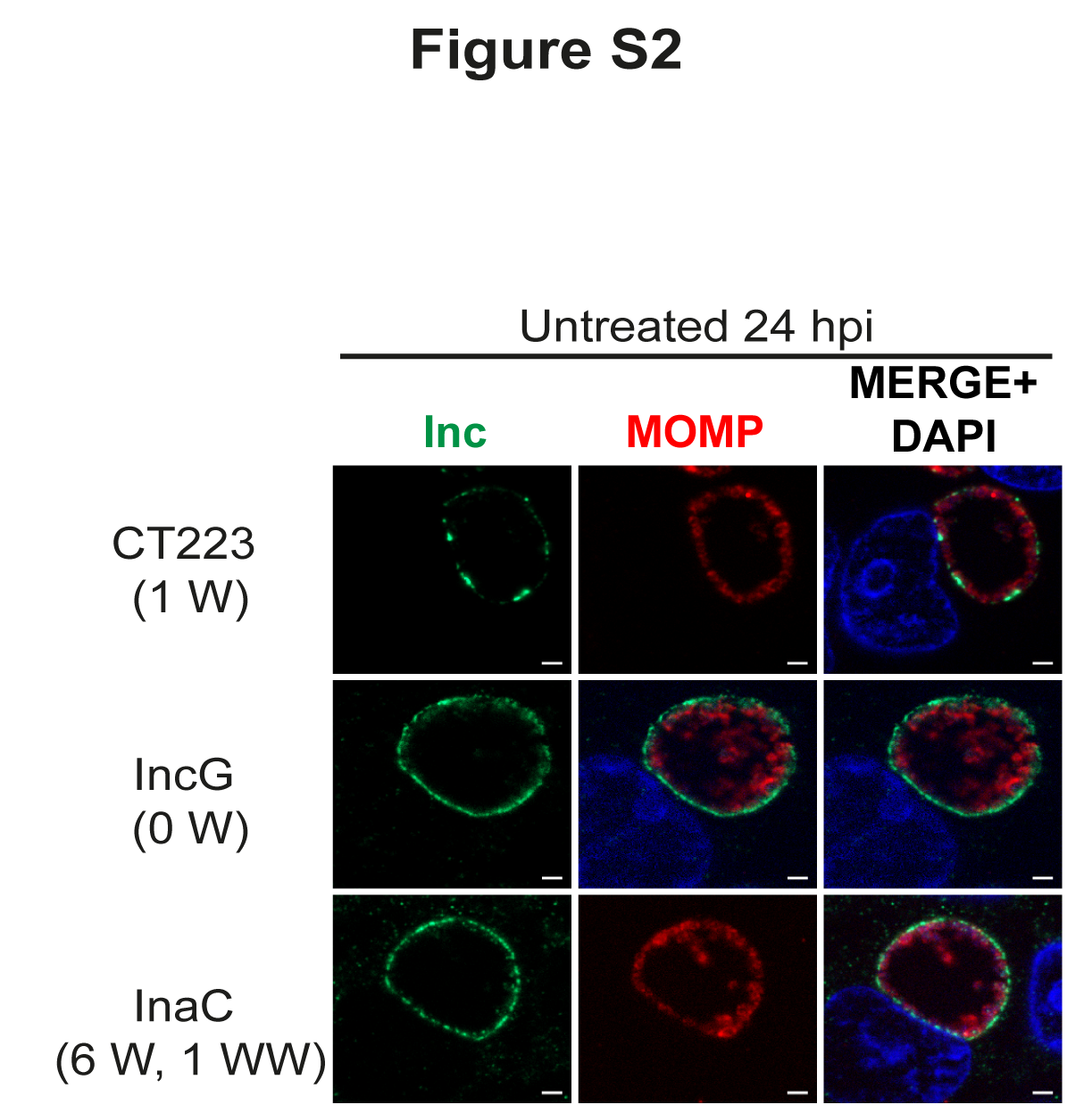

Supplement: Fig. S2 — Immunofluorescent images of CT223, IncG, and InaC at 24 hpi. [file iai.00532-24-s0002.tif]

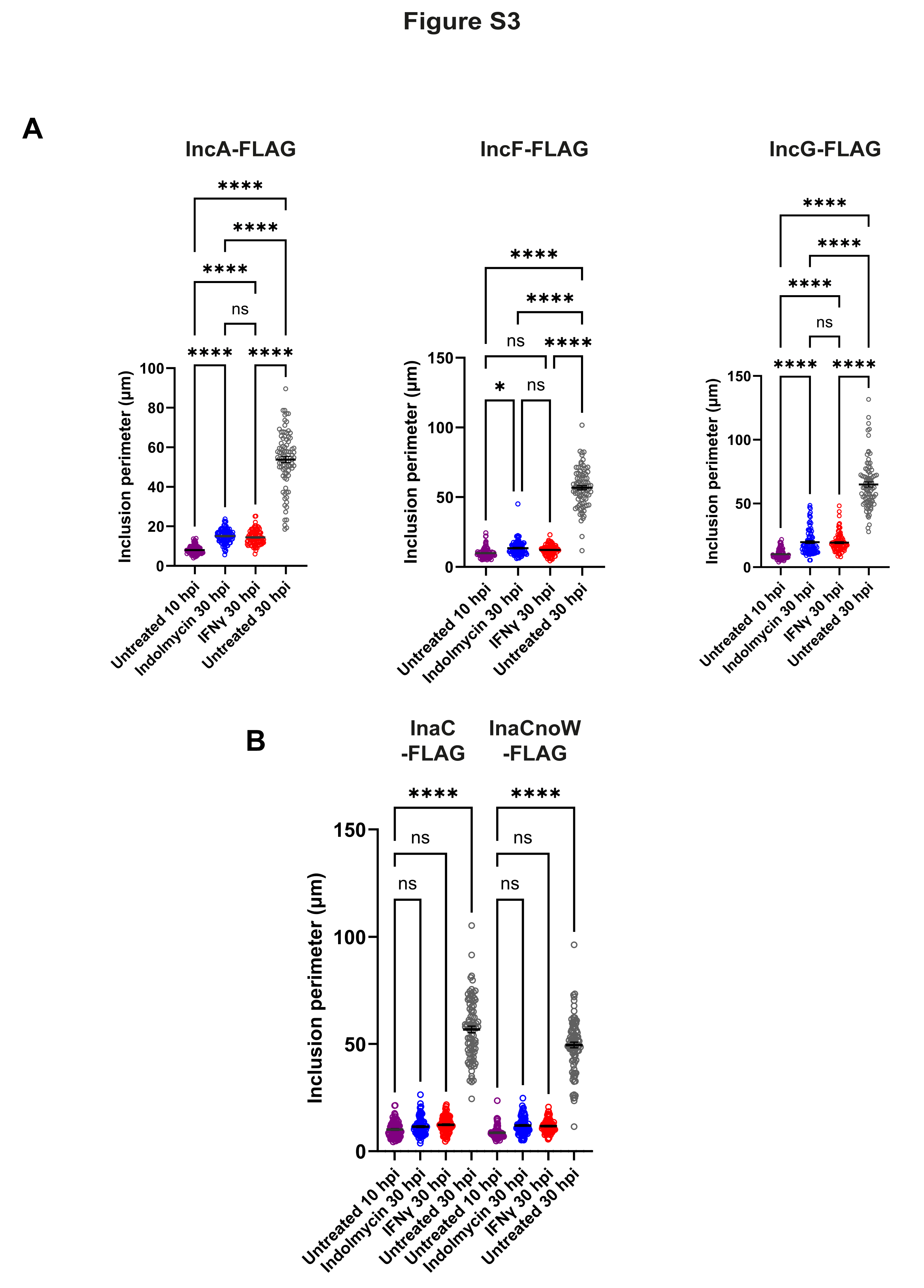

Supplement: Fig. S3 — Inclusion sizes corresponding to data in Fig. 5. [file iai.00532-24-s0003.tif]

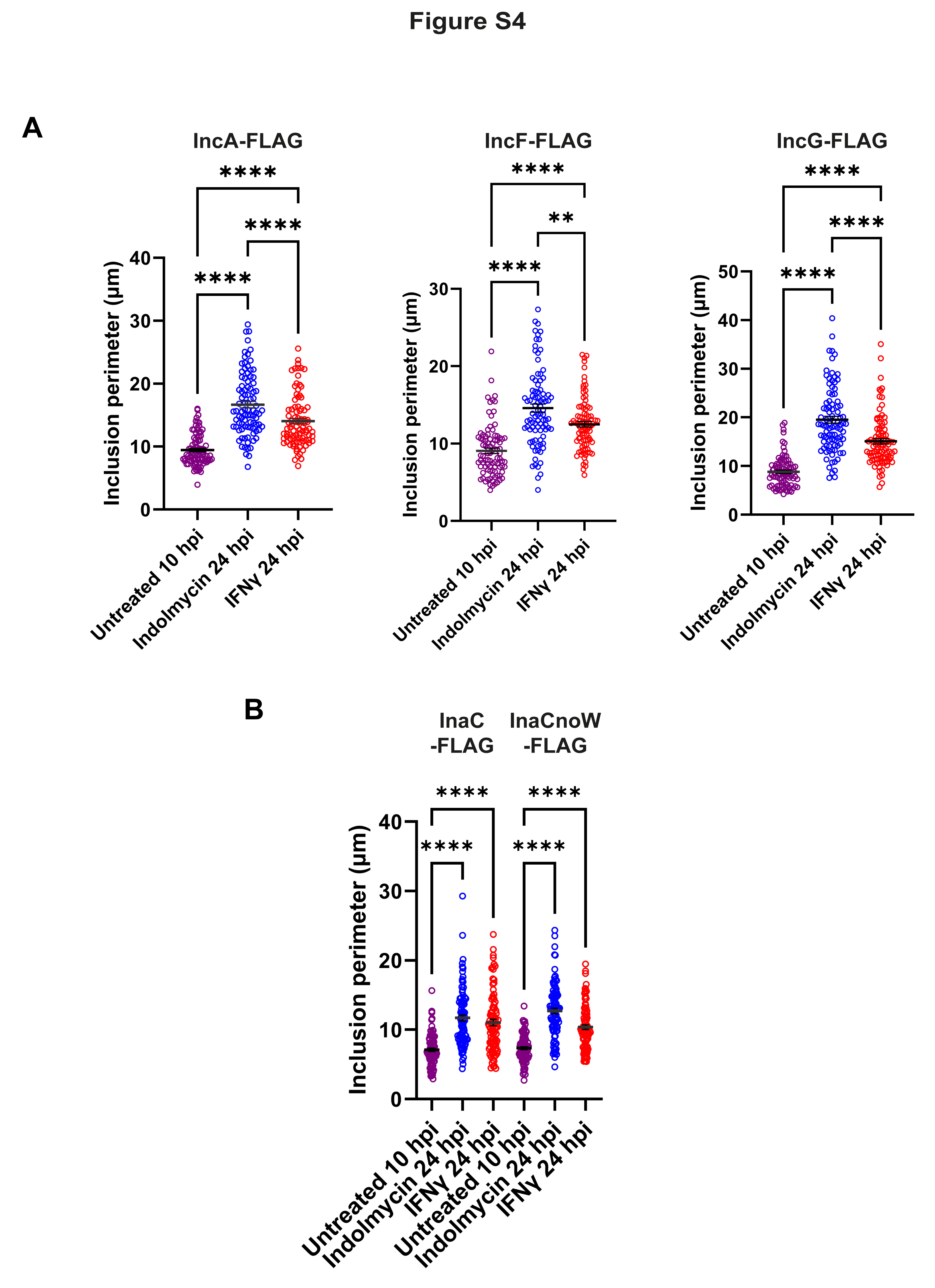

Supplement: Fig. S4 — Inclusion sizes corresponding to data in Fig. 6. [file iai.00532-24-s0004.tif]
