# Supplementary material for: Effect of tryptophan starvation on inclusion membrane composition and chlamydial-host interactions
Source: Infect Immun. 2025 Jan 13;93(2):e00532-24. doi: 10.1128/iai.00532-24 (PMC11834466; doi:10.1128/iai.00532-24)
Supplement: Supplemental material — Legends for associated supplemental tables and figures. [file iai.00532-24-s0005.docx]

**Supplemental Material**

**Supplementary Table 1. Tryptophan content of the T3S apparatus and chaperones in *Chlamydia trachomatis* L2.**

**Supplementary Table 2. List of Plasmids, Strains, and Primers used in this study.**

**Supplementary Figure 1. Diagrams and Inclusion Sizes related to Golgi trafficking experiments. (A, C)**A diagram that depicts the experimental design and includes important timepoints within the experiments.**(B)**The inclusion area (μm^2^) of the NBD-ceramide-labeled inclusions was quantified using Fiji/ImageJ software. The results were graphed and statistically analyzed using GraphPad Prism. Horizontal lines indicate means, and vertical lines indicate standard errors of the means. Statistical significance was determined by ordinary one-way ANOVA with Šidák’s multiple comparisons. **, p<0.01; ****, p<0.0001.**(D)**The inclusion perimeter (μm) was determined, and results were graphed and statistically analyzed as in **B**. Horizontal lines indicate means, and vertical lines indicate standard errors of the means. Statistical significance was determined by ordinary one-way ANOVA with Šidák’s multiple comparisons. ns, non-significant; ****, p<0.0001.

**Supplementary Figure 2. Localization of the CT223, IncG, and InaC Incs at the inclusion membrane during normal developmental cycle.**

HEp-2 cells were infected with *C. trachomatis* serovar L2 at an MOI of 1. Samples were fixed at 24 hpi and processed for indirect immunofluorescence to detect Incs (green), MOMP (red), and DNA (blue). Scale bar = 2 μm.

**Supplementary Figure 3. Persistence mediated by Trp-starvation negatively impacts inclusion growth compared to untreated control during Inc secretion experiments. (A, B)**Inclusion perimeters (μm) were quantified using Fiji/ImageJ software, and results were graphed and statistically analyzed using GraphPad Prism. Horizontal lines indicate means, and vertical lines indicate standard errors of the means. Statistical significance was determined by ordinary one-way ANOVA with Šidák’s multiple comparisons. ns, non-significant; *, p<0.05; ****, p<0.0001.

**Supplementary Figure 4. Persistence mediated by Trp-starvation negatively impacts inclusion growth compared to untreated control during Inc stability experiments. (A, B)** Inclusion perimeters (μm) were quantified using Fiji/ImageJ software, and results were graphed and statistically analyzed using GraphPad **P**rism. Horizontal lines indicate means, and vertical lines indicate standard errors of the means. Statistical significance was determined by ordinary one-way ANOVA with Šidák’s multiple comparisons. **, p<0.01; ****, p<0.0001.
