# Supplementary material for: Effect of tryptophan starvation on inclusion membrane composition and chlamydial-host interactions
Source: Infect Immun. 2025 Jan 13;93(2):e00532-24. doi: 10.1128/iai.00532-24 (PMC11834466; doi:10.1128/iai.00532-24)
Supplement: Table S1 — Tryptophan content of the chlamydial type III secretion system. [file iai.00532-24-s0006.pdf]

Supplementary Table 1. Tryptophan content of the T3S apparatus and chaperones in *Chlamydia trachomatis* L2.

Chlamydial T3S apparatus proteins			WW motif		
**L2/434/Bu**	**Common protein name**	**Proposed function**	**Amino acid length**	**Number of tryptophan (W)**	**% W**
CTL0038	CdsN	inner membrane ATP synthase, part of Hub complex	442	0	0.00
CTL0086	FliI	inner membrane ATP synthase, part of Hub complex	434	2	0.46
CTL0039	CdsO	stalk	168	1	0.60
CTL0824	CdsL	support proteins "spokes" inner membrane basal body	223	1	0.45
CTL0041	CdsQ	C-ring of inner membrane complex/cytoplasmic sorting platform	373	3	0.80
CTL0088	FliF	flagellar M-ring protein	334	1	0.30
CTL0316	CdsV	export gate	605	9	1.49
CTL0346	CdsU	inner membrane basal body component	360	1	0.28
CTL0825	CdsR	export apparatus	306	2	0.65
CTL0826	CdsS	export apparatus	94	2	2.13
CTL0827	CdsT	export apparatus	289	7	2.42
CTL0087	FliH	flagellar assembly protein; inner ring component	174	1	0.57
CTL0033	CdsD	inner membrane ring of basal body; orthologs: PrgH, MxiG	829	2	0.24
CTL0822	CdsJ	basal body component that spans periplasm	325	5	1.54
CTL0043	CdsC	outer membrane ring	921	5	0.54
CTL0035	CdsF	Needle protein, SctF	83	0	0.00
CTL0040	CdsP	molecular ruler	283	1	0.35
CTL0847	none	Needle tip; IpaD LcrV	183	0	0.00
CTL0841	CopB	needle tip; translocator	487	2	0.41
CTL0842	CopD	needle tip; translocator	439	2	0.46
CTL0236	CopB2	needle tip; translocator	506	7	1.38
CTL0235	CopD2	needle tip: translocator	493	3	0.61
CTL0655	CdsZ	FlgZ protein; flagellar-associated zinc-ribbon domain protein	254	1	0.39
CTL0553	PknD	serine/threonine kinase targets CdsD	934	13	1.39
CTL0345	LcrD	low calcium sensor	708	5	0.71
CTL0344	CopN	low calcium response protein	421	0	0.00
		**Totals**	10668	76	0.71
		**Averages**	410.31	2.92	0.70

Chlamydial T3S chaperone proteins					
L2/434/Bu	Common protein name	Class	Amino acid length	Number of tryptophan (W)	% W
CTL0343	Scc1	IA	146	1	0.68
CTL0032	Scc4	IB	133	1	0.75
CTL0299	Slc1	IB	167	1	0.60
CTL0512	Mcsc	IB	163	1	0.61
CTL0847	unassigned	IB	183	0	0.00
CTL0839	Scc2	II	232	0	0.00
CTL0526	unassigned	II	139	2	1.44
CTL0237	Scc3	II	198	3	1.52
CTL0034	CdsE	III/ *V*?	83	0	0.00
CTL0036	CdsG	III	149	2	1.34
CTL0369	unassigned	unknown, likely II; TPR domain: aa261-462	486	2	0.41
		**Totals**	**2079**	**13**	**0.63**
		**Averages**	**189**	**1.18**	**0.67**

	Amino acid length	trp	% W
**Total apparatus + chaperones**	**12747**	**89**	**0.70**
**Average apparatus + chaperones**	**344.51**	**2.41**	**0.69**

Unknown possible T3S associated protein			
L2/434/Bu	Amino acid length	Number of tryptophan (W)	% W
CTL0238	482	3	0.62
