# Supplementary material for: Effect of tryptophan starvation on inclusion membrane composition and chlamydial-host interactions
Source: Infect Immun. 2025 Jan 13;93(2):e00532-24. doi: 10.1128/iai.00532-24 (PMC11834466; doi:10.1128/iai.00532-24)
Supplement: Table S2 — List of plasmids, strains, and primers used in this study. [file iai.00532-24-s0007.docx]

| **Construct Plasmid** | **Relevant genotype** | **Ori** | **Source of Reference** |
| --- | --- | --- | --- |
| pBOMB4-Tet | *bla* P*tet* | pUC19 | (1) |
| pBOMB_*incA*-FLAG | *bla* P*_tet_*::*incA*-FLAG | pUC19 | (2) |
| pBOMB-*ct813*-FLAG | *bla* P*_tet_*::*ct813*-FLAG | pUC19 | (3) |
| pBOMB-*ct813noW*-FLAG | *bla* P*_tet_*::*ct813noW*-FLAG | pUC19 | This study |
| pBOMBL | *bla* P*tet* | pUC19 | (4) |
| pBOMBL-*incF*-FLAG | *bla* P*_tet_*::*incF*-FLAG | pUC19 | This study |
| pBOMBL-*incG*-FLAG | *bla P_tet_::incG*-FLAG | pUC19 | This study |
| pBOMBmC | *bla* P_Nm_::*mCherry* | pUC19 | (5) |
| pBOMBmC-*gfp(ssrA_VDD)* | *bla* P_Nm_::*mCherry* P*_tet_*::*gfp(ssrA_VDD)* | pUC19 | (5) |

**Supplementary table 1. The list of Plasmids, Strains and Primers used in this study.**

| ***E. coli* Strain** | **Relevant genotype** | **Source of Reference** |
| --- | --- | --- |
| DH10β | *Δ(ara-leu) 7697 araD139 fhuA ΔlacX74 galK16 galE15 ϕ80dlacZΔM15 (e14-) recA1 relA1 endA1 nupG rpsL (*Str^R^*) rph spoT1 Δ(mrr-hsdRMS-mcrBC)* | New England BioLabs |

| **Primer name** | **Sequence** | **Features** | **Usage** |
| --- | --- | --- | --- |
| *incF* 5' LIC F-pBOMBL | AAAGATCTTCACACAGGACATCTGCATGGGAGACGTGATGATAC | 5' end overlaps pBOMBL at EagI site | For *incF*-FLAG insertion into pBOMBL |
| *incF* 3' LIC R-Flex Linker | ACTTCCTCCCCCAGAACCGCCACCGCACTTATTTGTAGAAGC | 3' end overlaps with linker | For *incF*-FLAG insertion into pBOMBL |
| *incG*-pBOMBL_EagI_5' LIC Fwd | AAAGATCTTCACACAGGACATCTGCATGATCTGTTGTGACAAAGTCTTG | 5' end overlaps pBOMBL at EagI site | For *incG*-FLAG insertion into pBOMBL |
| *incG*-FlexLinker_EagI_3' LIC Rev | ACTTCCTCCCCCAGAACCGCCACCGAAGGAGCGTGATCGAGAAC | 3' end overlaps with linker | For *incG*-FLAG insertion into pBOMBL |

| **gBlock name** | **Sequence** | **Features** | **Usage** |
| --- | --- | --- | --- |
| *ct813noW*-FLAG_gBlock | aaagaggagaaaggatctgcATGACTACTCTTCCCAATACTTGTACTTCAAACTCCAATTCTATAAATACTTTCACGAAAGATATCGAAATGGCAAAGCAGATCCAAGGCTCTCGGAAGGATCCTCTTGCTAAAACCTCA**TTT**ATCGCAGGATTGATCTGTGTTGTTGCTGGGGTATTGGGACTTCTTGCTATAGGAATAGGAGGATGTTCTATGGCTTCCGGATTAGGATTAATCGGTGCTATAATTGCTGCTGTCGTCGTAGCCGTCGGTTTATGCTGCTTGGTATCAGCTTTATGCTTGCAAGTTGAGAAATCTCAA**TTTTTC**CAGAAAGAGTTTAAGTCT**TTT**ATAGAGCAAAAATCCCAGTTTAGAATTGTAATGGCTGACATGCTCGAAGCAAATCAGAAATTGCAATCAGAAGTGGAATTCCTTTCAAAAGGT**TTT**AGCGATGCTGCCGCAGTGCACAAGGAAGATGTGACGAAGTATGAGCAAGTTGTTGAAAAATATGGTGAGAAAATTATGAAGCTATATAAGCAAACAGGTGTTCTTACCATAGAAAAAGTCAATCTTCAGAAAGAGAAAAAAACT**TTT**CTGGAAGAGAAAGCTGAAATGGAGCAAAAATTAACAACCGTTACGGATCTGGAGGCAGCAAAACAACAATTAGAAGAGAAAGTGACAGATCTAGAGTCAGAGAAACAAGAGTTGCGTGAAGAATTAGACAAAGCTACAGAGAATCTTGATGAGATGGCTCATGAAGCGATGGAATTCGAAAAAGAAAAACATGGTATCAAACCAGGAAGACGTGGTTCGATAGACTACAAGGATGACGACGATAAGTAAgtaccggtcgaccattcaaa | Lowercase represents pBOMB4 flanking regions for Hifi insertion and underline represents the FLAG tag. Bolded sequences indicate mutations in order to replace tryptophan codons by phenylalanine codons. | Template for Hifi reaction and *ct813noW*-FLAG into pBOMB4 |
| *pBOMBL-FlexLinker +Flag*_gBlock | CAAAAGATCTTCACACAGGACATCTGCGGCCggtggcggttctgggggaggaagtGACTACAAAGACGATGACGACAAGTAGTCGACCATTCAAATATGTATCCGCTCATGAGACAATAACCCTG | Lowercase represents the flexible linker and underline represents the FLAG tag. | Template to insert a flexible glycine-glycine-glycine-serine (repeated twice) linker and FLAG tag between the EagI and SalI sites of pBOMBL |

1. Bauler, L.D. and Hackstadt, T. (2014) Expression and targeting of secreted proteins from Chlamydia trachomatis. *J Bacteriol*, **196**, 1325-1334.

2. Bui, D.C., Jorgenson, L.M., Ouellette, S.P. and Rucks, E.A. (2021) Eukaryotic SNARE VAMP3 Dynamically Interacts with Multiple Chlamydial Inclusion Membrane Proteins. *Infect Immun*, **89**.

3. Olson-Wood, M.G., Jorgenson, L.M., Ouellette, S.P. and Rucks, E.A. (2021) Inclusion Membrane Growth and Composition Are Altered by Overexpression of Specific Inclusion Membrane Proteins in Chlamydia trachomatis L2. *Infect Immun*, **89**, e0009421.

4. Ouellette, S.P., Blay, E.A., Hatch, N.D. and Fisher-Marvin, L.A. (2021) CRISPR Interference To Inducibly Repress Gene Expression in Chlamydia trachomatis. *Infect Immun*, **89**, e0010821.

5. Wood, N.A., Blocker, A.M., Seleem, M.A., Conda-Sheridan, M., Fisher, D.J. and Ouellette, S.P. (2020) The ClpX and ClpP2 Orthologs of Chlamydia trachomatis Perform Discrete and Essential Functions in Organism Growth and Development. *mBio*, **11**.
